# Comparative outcome analysis of bleb needling of fibrotic blebs in the clinic versus the operating room: a retrospective case series

**DOI:** 10.1186/s12886-021-01870-1

**Published:** 2021-03-04

**Authors:** Vikram Ponnusamy, Van Nguyen, Jella A. An

**Affiliations:** 1 University of Missouri School of Medicine, Columbia, MO USA; 2 Department of Ophthalmology, Mason Eye Institute, University of Missouri Health Care, 1 Hospital Drive, Columbia, MO 65201 USA

**Keywords:** Trabeculectomy, ExPress shunt, XEN gel stent, Bleb needling, Bleb revision

## Abstract

**Background:**

To compare 6 month outcomes of bleb needling performed in the clinic vs. the operating room (OR) in adult glaucoma patients with failed bleb.

**Methods:**

A retrospective case series of 47 eyes from 41 glaucoma patients who received needling with mitomycin C (MMC) of scarred bleb from prior bleb-forming procedures in clinic (32 eyes) vs. the OR (15 eyes), including trabeculectomy (14 eyes), ExPress shunt (16 eyes), and ab-interno XEN gel stent (17 eyes). The primary outcome was needling success, defined as IOP ≤ 18 mmHg on 0 glaucoma medications without requiring an additional IOP lowering procedure within 6 months after needling.

**Results:**

At 6 months, bleb needling success rate was similar when performed in the clinic vs. in the OR (28% vs. 20%, *P* = 0.54). Success rate was not statistically different in patients with prior trabeculectomy, ExPress shunt, and XEN gel stent (29% vs. 38% vs. 12%, *P* = 0.26). When comparing clinic vs. the OR needling procedures at 6 months, there was no difference in mean IOP (14.2 vs. 14.9 mmHg, *P* = 0.73), mean glaucoma medications (1.4 vs. 1.7, *P* = 0.69), additional IOP-lowering procedure rate (16% vs. 27%, *P* = 0.37), or complication rate (0% vs. 7%, *P* = 0.32).

**Conclusion:**

Bleb needling with MMC in clinic may be a safe and effective way to revise failed bleb after trabeculectomy, ExPress shunt, and XEN gel stent procedures when compared to needling in the OR.

**Supplementary Information:**

The online version contains supplementary material available at 10.1186/s12886-021-01870-1.

## Introduction

Glaucoma filtration surgery is a bleb-forming procedure that effectively lowers intraocular pressure (IOP). We now have a number of ways to perform bleb-forming filtration surgery. Trabeculectomy is one of the earliest and most frequently performed glaucoma surgeries worldwide since 1968 [1, 2]. ExPress shunt (Alcon Laboratories, Fort Worth, TX, USA) was found to result in comparable but safer surgical outcomes by increasing reproducibility and preventing intraoperative anterior chamber collapse [3]. Most recently, the XEN gel stent (Allergan PLC, Irvine, CA, USA) was developed to enable bleb formation via an ab-interno approach using a collagen stent with a 45 μm inner-diameter. As a result, XEN gel stent is shown to significantly decrease the risk of hypotony, duration of surgery, recovery time, and improve reproducibility of bleb formation [4]. Each of these procedures relies upon a bleb to filter out fluid from the anterior chamber to control IOP.

Early or late fibrosis of a bleb may decrease efficacy of the filtration channel, leading to suboptimal IOP lowering and eventual failure of filtration surgery. Bleb needling with or without antifibrotic drugs is a relatively safe and effective way to remove scar tissue and improve filtration, and it can easily be done in the clinic setting on a slit lamp with few long term complications and prevent re-operation [5, 6]. Bleb needling involves cutting through fibrotic tissue in the bleb, which reopens the channel. The concurrent use of antifibrotic medications in this procedure helps to prevent the recurrence of fibrosis by decreasing fibroblast production and scar tissue formation. However, theoretical risks of bleb needling, including bleb leaks, choroidal effusions, suprachoroidal hemorrhage, and endophthalmitis, sway many ophthalmologists to perform bleb needling exclusively in the operating room or avoid the procedure all together.

Studies evaluating success of bleb needling for trabeculectomy, ExPress shunt, and XEN gel stent show up to 40% success [7–9]. However, a number of studies evaluating the clinical outcomes after placement of a XEN gel stent report higher early postoperative bleb needling rates up to 44.1% at 1 year [4, 10–12]. The alternative options to bleb-needling are reintroducing glaucoma medications and undergoing further laser procedures or surgeries, which may not be in the patient’s best interest. Though bleb needling is a procedure that has been shown to be effective, there are no studies that compare whether bleb needling performed in clinic is as successful and as safe as bleb needling done in the operating room (OR) in different types of bleb-forming procedures.

In this study, we compared 6 month outcomes after bleb needling procedures performed in the clinic or the OR for failed blebs after trabeculectomy, ExPress shunt, and XEN gel stent procedures in adult glaucoma patients.

## Materials and methods

Institutional Review Board (IRB) approval was obtained from the University of Missouri to conduct a retrospective chart review of 47 eyes of 41 adult glaucoma patients who underwent bleb needling procedures at Mason Eye Institute between January 1, 2016 and October 31, 2018 (IRB# 2012046). Inclusion criteria was poorly controlled IOP with or without medications and evidence of bleb fibrosis in patients with prior trabeculectomy, ExPress shunt, or XEN gel stent procedures, with minimum 6 months follow up after bleb needling. All procedures were performed by a single surgeon. All patients received 0.02–0.04 mg of mitomycin (MMC) subconjunctival injection prior to needling, and prior ExPress shunt patients received YAG laser to the inner lumen of the shunt prior to bleb needling. Exclusion criteria were any patients who received any other procedure (Kahook Dual Blade goniotomy, MicroPulse cyclophotocoagulation, Ahmed Valve placement) at the time of bleb needling. Baseline characteristics, visual acuity (VA), IOP, and number of glaucoma medications were obtained at the time of needling, at 1 week, 1 month, 2 months, and 6 months postoperatively. Location (clinic vs. the OR), additional IOP-lowering procedures required (including repeat bleb needling) within 6 months, and any adverse events were also recorded. A de-identified data set was analyzed.

The primary outcome was comparing bleb needling success in clinic vs. the OR, where success was defined as IOP ≤ 18 mmHg, on 0 glaucoma medications, and no additional IOP lowering procedure within 6 months of needling. An eye with any vision-threatening complication was considered to be failure. Secondary subgroup analyses included bleb needling success, mean IOP, mean glaucoma medications, complications, additional IOP-lowering procedures, and re-needling rate (defined as repeat needling procedures performed within 3 months of the most recent needling) between trabeculectomy, ExPress shunt, and XEN gel stent groups. Vision-threatening complications were defined as any complication leading to loss of two or more Snellen lines.

### Statistical analysis

A priori power analysis with a goal power of 80% and effect size of 0.5 suggested a total goal sample size of 32 in comparing clinic vs. the OR needling. χ^2^-test and Fisher’s exact tests were used to compare success between these subgroups. ANOVA and paired *t*-tests were used to compare percent IOP reduction and glaucoma medication reduction. Kaplan-Meier survival analysis and log-ranked test were performed to assess bleb survival. All statistical analysis and multinomial logistic modeling were performed using SPSS Statistics (IBM Corporation; Armonk, NY, USA).

### Ocular physical examination

Visual acuity was measured by a technician in clinic with a patient reading a multi-letter Snellen chart from 20 ft away with one eye at a time. IOP was measured by an ophthalmologist or a trained technician by utilizing a Goldmann applanation tonometer. The anterior segment and posterior segment exams were done by an ophthalmologist utilizing a slit lamp microscope. Patients were dilated for indirect ophthalmoscopy if there is an indication suggesting a posterior segment pathology.

### Bleb needling procedure

The eye was prepped with moxifloxacin in clinic and povidone in the OR. For both locations, topical tetracaine drop and lidocaine gel were used for local anesthesia, and mild intravenous sedation was administered for those treated in the OR. All patients received Mitomycin-C (0.1–0.2 mL of 0.2 mg/mL) injected subconjunctivally with a 30 gauge needle at the site of bleb needling, massaged with a cotton-tip applicator to distribute evenly away from the limbus, and left for over 20 min to bind. All needling performed in clinic was done under a slit lamp microscope. A 27 or 30 gauge needle on a 1 mL syringe with air was used to release subconjunctival scar tissues. In failed trabeculectomies, the scleral flap was lifted with the needle and air was injected into the anterior chamber as needed to keep the anterior chamber from collapsing. In failed ExPress Shunts, one to two 8 mJ shots of YAG laser was directed at the internal ostium visualized by gonioscopy to clear any debris within the shunt prior to needling. In failed XEN gel stents, a 30 gauge needle was used to gently remove any subconjunctival and Tenon adhesions above and beneath the subconjunctival side of the stent; in addition, a dispersive viscoelastic was injected into the subconjunctival space as a spacer and to tamponade any heme as needed. If done in the OR, a Blumenthal conjunctival dissector was used to further release any posterior subconjunctival adhesion and the entry was sutured with 9–0 Vicryl.

## Results

### Baseline characteristics

The average age of patients was 71.6 ± 8.6 (range, 51 to 84), with females comprising 40.4% (19/47 eyes). The majority of patients were Caucasian (85.1%). Preoperative demographic and glaucoma information is reported in Table 1.

**Table 1:** Baseline demographic data

	Overall (***n*** = 47)	Clinic (***n*** = 32)	OR (***n*** = 15)	
Age (years), mean ± SD	71.6 ± 8.6	73.4 ± 7.8	68.0 ± 9.2	
Female Gender, n (%)	19 (40.4)	15 (46.9)	4 (26.7)	
Ethnicity, n (%)				
Caucasian	40 (85.1)	27 (84.4)	13 (86.7)	
Black	6 (12.8)	5 (15.6)	1 (6.7)	
Asian	1 (2.1)	0 (0)	1 (6.7)	
Type of Initial Procedure, n (%)				
Trabeculectomy	14 (29.8)	6 (18.8)	8 (53.3)	
ExPress	16 (34.0)	12 (37.5)	4 (26.7)	
XEN	17 (36.2)	14 (43.8)	3 (20.0)	
Time Until Needling (months), mean ± SD				
Trabeculectomy	77.4 ± 48.1	77.6 ± 54.8	77.2 ± 50.5	*P* = 0.992
ExPress	39.4 ± 24.7	39.5 ± 22.3	39.2 ± 34.1	*P* = 0.986
XEN	1.7 ± 1.7	1.8 ± 1.9	1.6 ± 0.8	*P* = 0.877
	*P* < 0.001*	*P* < 0.001*	*P* < 0.001*	
Baseline IOP (mmHg), mean ± SD	24.2 ± 8.5	23.3 ± 9.1	26.1 ± 6.8	*P* = 0.243
Trabeculectomy	25.1 ± 8.4	25.3 ± 11.1	24.9 ± 6.5	*P* = 0.931
ExPress	21.6 ± 5.1	19.7 ± 3.7	27.3 ± 4.6	*P* = 0.035*
XEN	26.0 ± 10.7	25.6 ± 10.9	28.0 ± 11.3	*P* = 0.757
	*P* = 0.297	*P* = 0.220	*P* = 0.764	
Baseline medications, mean ± SD	1.9 ± 1.6	2.0 ± 1.7	1.9 ± 1.5	*P* = 0.835
Trabeculectomy	2.5 ± 1.6	3.7 ± 0.8	1.6 ± 1.4	*P* = 0.006*
ExPress	2.3 ± 1.6	2.0 ± 1.7	3.3 ± 1.0	*P* = 0.096
XEN	1.1 ± 1.4	1.2 ± 1.4	0.7 ± 1.2	*P* = 0.521
	*P* = 0.025*	*P* = 0.006*	*P* = 0.051	

### Location of needling: in clinic vs. in the OR

**Fig. 1:**
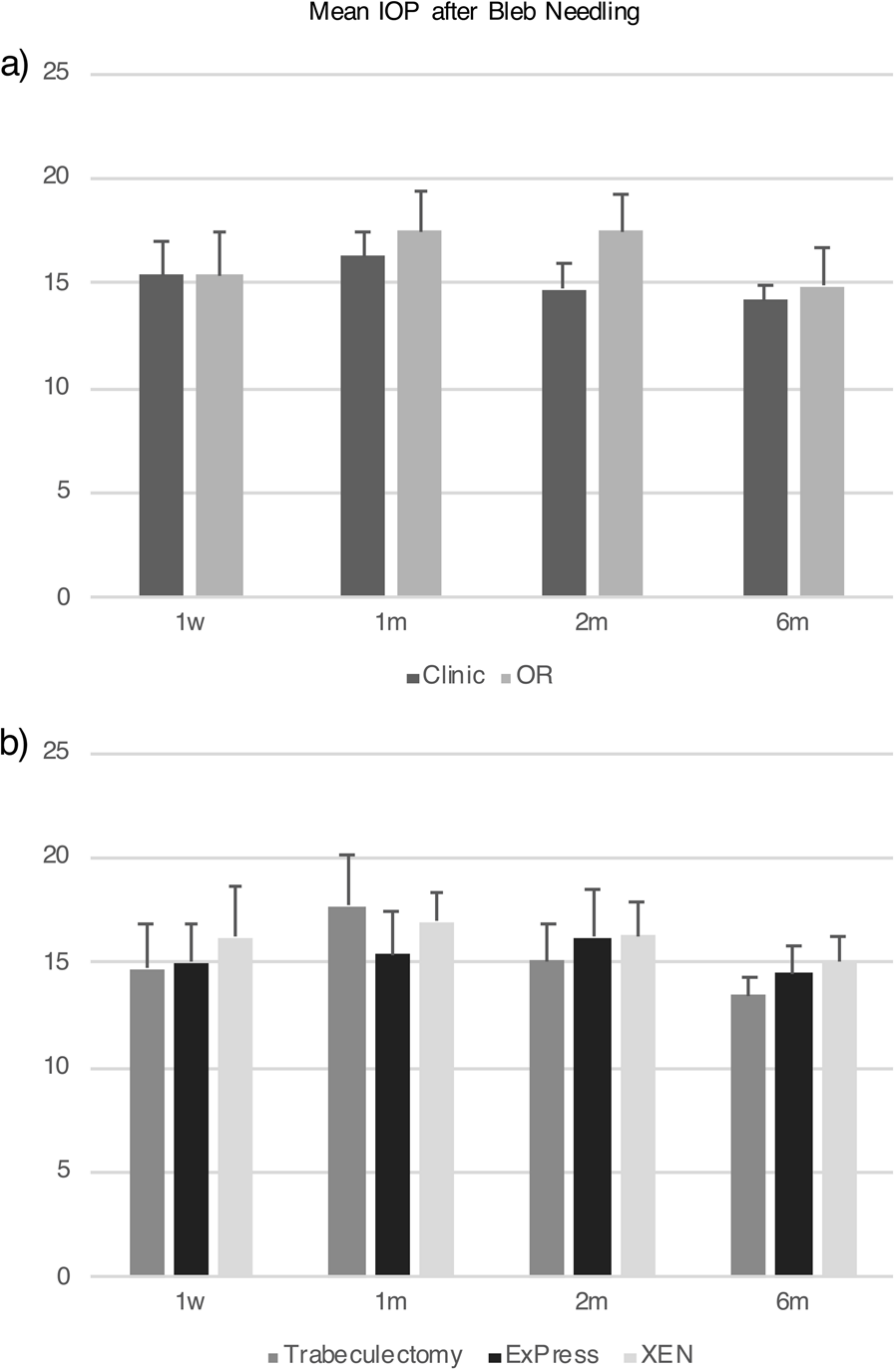
There is no difference in mean IOP for bleb needling performed in the clinic vs. the OR at any time point (**a**) or performed after trabeculectomy, ExPress shunt, or XEN gel stent procedure at any time point (**b**)

**Fig. 2:**
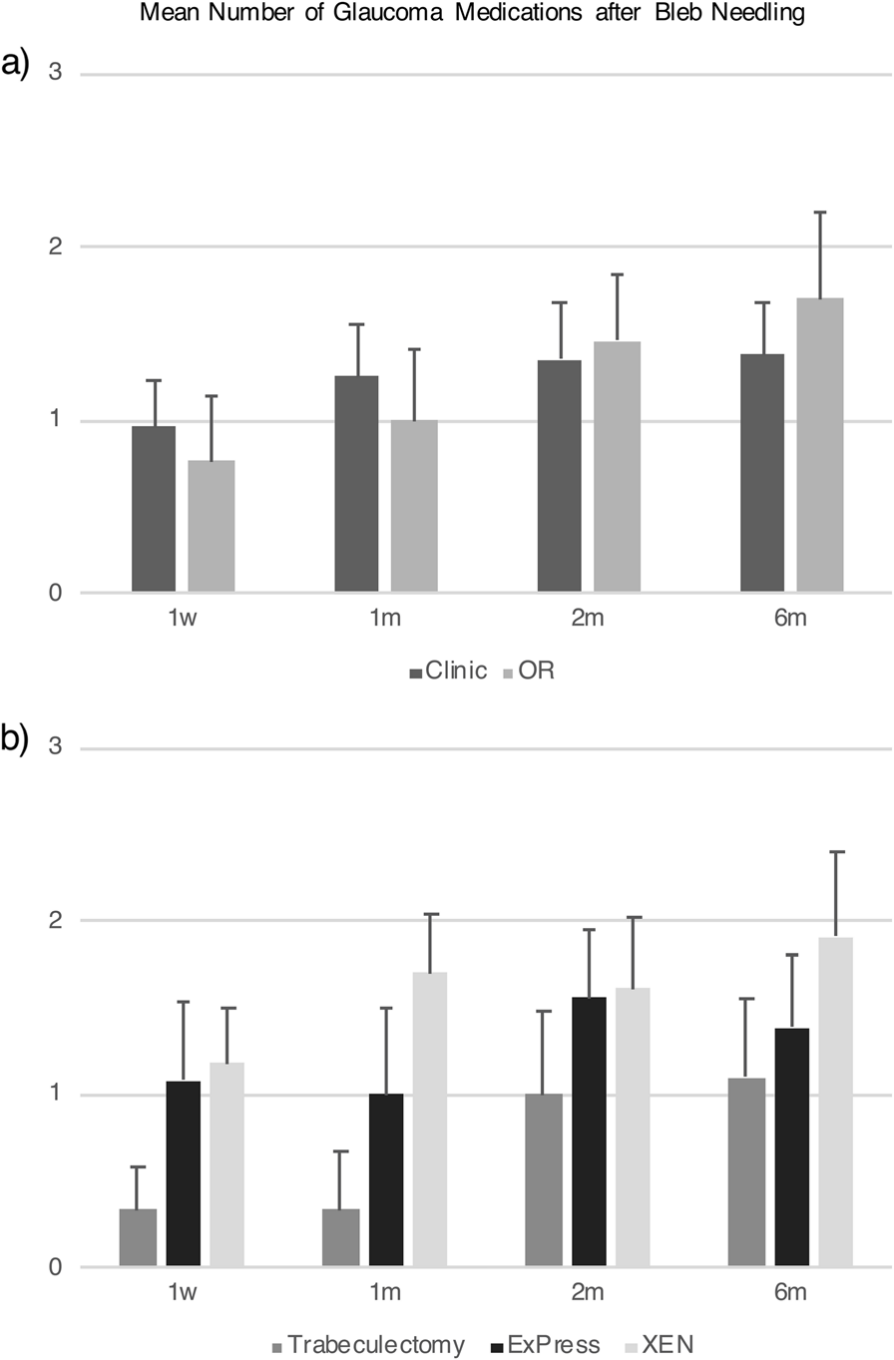
There is no difference in mean number of glaucoma medications for bleb needling performed in the clinic vs. the OR at any time point (**a**), or performed after trabeculectomy, ExPress shunt, or XEN gel stent procedure at any time point (**b**)

Of all bleb needling procedures performed, 68.0% were in clinic and 31.9% were in the OR (Table 1). Baseline IOP was 23.3 ± 9.1 mmHg and 26.1 ± 6.8 mmHg for patients with bleb needling performed in clinic vs. the OR, respectively (*P* = 0.24). Mean baseline number of glaucoma medications was 2 classes for needling in clinic and in the OR (*P* = 0.84, Table 1). In-clinic needling tended to be more successful compared to in-OR needling (28% vs. 20% in the OR), although this was not statistically significant at any time point (*P* = 0.27 at 6 months; Table 2). Kaplan-Meier survival analysis with log rank test showed that there was no difference in survival of success between clinic vs. the OR (*P* = 0.82). There were also no differences in repeat needling success rate (Supplemental Table 1), mean IOP and number of medications (Fig. 1, Fig. 2, Supplemental Table 2), and complication rate (0% vs. 7%, *P* = 0.32) between needling performed in clinic vs. the OR within 6 months. There was no difference in bleb needling patients requiring an additional IOP lowering procedure within 6 months (15.6% vs. 26.7%, *P* = 0.37, Table 3).

**Table 2:** Bleb revision success rate at each time point

	1w	1 m	2 m	6 m
Clinic	43.8% (14/32)	31.3% (10/32)	31.3% (10/32)	28.1% (9/32)
OR	53.3% (8/15)	40% (6/15)	20% (3/15)	20% (3/15)
*P* value	0.539	0.555	0.422	0.552
Trabeculectomy	64.3% (9/14)	50% (7/14)	28.6% (4/14)	28.6% (4/14)
ExPress	43.8% (7/16)	37.5% (6/16)	37.5% (6/16)	37.5% (6/16)
XEN	35.3% (6/17)	17.6% (3/17)	17.6% (3/17)	11.8% (2/17)
P value	0.261	0.157	0.442	0.227

Abbreviations: *w* week, *m* month(s)

**Table 3:** Additional Procedure Rate^a^

Clinic	15.6% (5/32)
OR	26.7% (4/15)
*P* value	0.370
Trabeculectomy	42.9% (6/14)
ExPress	25.0% (4/16)
XEN	29.4% (5/17)
*P* value	0.746

^a^Proportion of patients that required an additional bleb needling or other IOP lowering procedure (cataract extraction, MicroPulse transscleral cytophotocoagulation, Ahmed valve, or Kahook dual blade goniotomy) within 6 months

### Trabeculectomy vs. ExPress shunt vs. XEN gel stent

**Fig. 3:**
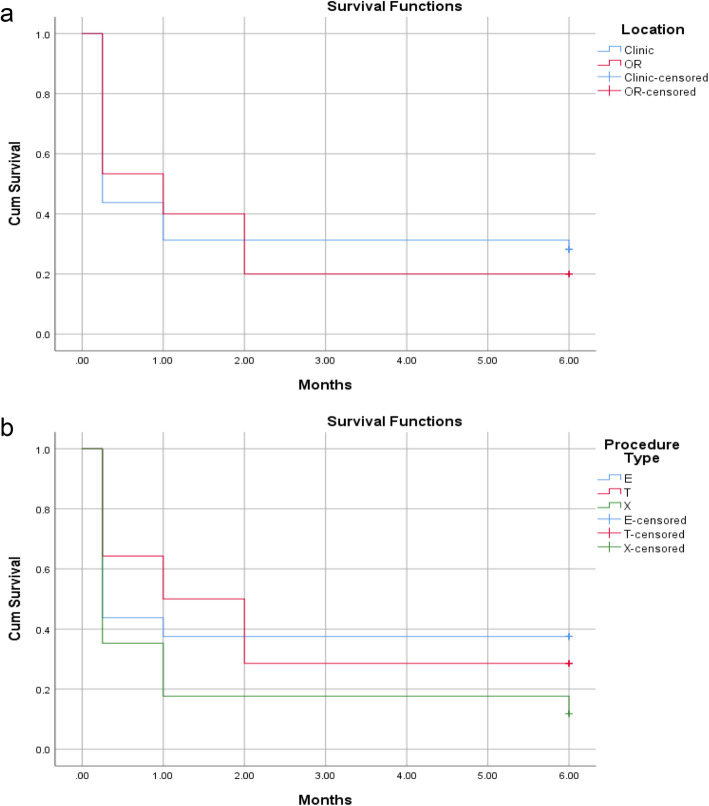
There is no difference in Kaplan-Meier survival of success for bleb needling performed in the clinic vs. the OR (*P* = 0.82, **a**) or after trabeculectomy vs. ExPress shunt vs. XEN gel stent (*P* = 0.23, **b**)

Fourteen patients (29.8%) had trabeculectomy, 16 patients (34.0%) had ExPress shunt, and 17 patients (36.2%) had XEN gel stent. The average time from initial surgery to bleb needling ranged from 15 days to 12 years. However, the time to needling was significantly less (*P* < 0.001) for XEN gel stent (1.7 ± 1.7 months) compared to trabeculectomy (77.4 ± 48.1 months) and ExPress shunt (39.4 ± 24.7 months). XEN gel stent patients were also on significantly fewer medications prior to undergoing needling (*P* = 0.03, Table 1). There was no statistically significant difference in success rate between bleb needling of trabeculectomy, ExPress shunt, or XEN gel stent procedures at any time point (29% vs. 38% vs. 12% at 6 months, *P* = 0.23; Table 2 and Fig. 3). Kaplan-Meier survival analysis with log rank test also showed that there was no difference in survival of success between trabeculectomy vs. ExPress vs. XEN (*P* = 0.23). There were no differences in mean IOP or mean number of medications between the three procedures at any time point (Supplemental Table 2).

### Multinomial logistic regression predicting 6 month success

A multinomial logistic regression of 6 month bleb needling success including bleb needling location, initial procedure type, baseline IOP, and baseline number of glaucoma medications showed no variables were significantly associated with an increase or decrease in the odds ratio of success (Supplemental Table 3).

### Re-needling rate

The re-needling rate was defined as the number of patients that required needling procedures performed within 3 months of the most recent needling, and it was calculated for each group (Supplemental Table 4). Overall, 25% of clinic and 7% of the OR needling procedures were repeat needling procedures (*P* = 0.136). The re-needling rate was 22, 13, and 18% for trabeculectomy, ExPress shunt, and XEN gel stent, respectively (*P* = 0.92).

### Complications

Only one bleb needling patient had a vision-threatening complication. This 81-year-old female patient had a kissing suprachoroidal hemorrhage 4 days after a bleb needling in the OR after failed trabeculectomy and required urgent drainage. Two months after drainage, her vision improved to 2 lines less than her preoperative best corrected VA due to development of chronic cystoid macular edema and epiretinal membrane. At this time point IOP was 7 mmHg on 0 medications. There were no other vision-threatening complications including endophthalmitis, hypotony, subconjunctival hemorrhage, shallow anterior chamber, peripheral choroidal detachments, or other complication that led to a vision loss greater than 2 Snellen lines.

## Discussion

Our study compared the success (defined as IOP ≤ 18 mmHg on 0 glaucoma medications and without requiring an additional IOP lowering procedure within 6 months) after bleb needling performed in the clinic vs. the OR for prior trabeculectomy, ExPress shunt, or XEN gel stent. Success rate, mean IOP, and mean number of medications was similar in patients who received needling in clinic vs. the OR (*P* > 0.05). These results suggest that outcomes after bleb needling in the clinic vs. the OR are equivalent, and they highlight the lack of benefit to performing a more costly version of the same procedure in the OR. Bleb needling performed in the clinic has a number of desirable advantages for patients, including decreased health care costs and a faster procedure time. Surgeons would also have the opportunity to treat a fibrotic bleb immediately in clinic rather than spending time and healthcare resources to schedule a patient in the OR. However, increased patient comfort with sedation in the OR is a key benefit that may influence patient-physician decision making. Our study found an overall success rate for bleb needling to be 25.5% at 6 months (28.1% in clinic vs. 20% in the OR). Thus, bleb needling may limit the necessity for further treatment with glaucoma drops or additional IOP lowering surgery in some patients. This makes it a worthwhile surgery salvaging technique, especially when done in clinic. The theoretical advantage of performing bleb needling in the OR would be a safer and more controlled environment. However, in our study, vision-threatening complications were rare, and it only occurred in one patient who received needling in the OR. This patient had a history of successful bleb needling of a failed trabeculectomy in clinic in the fellow eye, and from a baseline IOP of 19 mmHg on 4 classes of medications she achieved 10 mmHg off all medications postoperatively. The patient however preferred needling of the other eye in the OR under sedation to reduce discomfort. The procedure was unremarkable, and the IOP was successfully lowered from 21 mmHg on 4 classes to 4 mmHg off all medications on postoperative day 1. The patient returned back to emergency clinic on day 4 with sudden loss of vision in this eye, and she was found to have kissing suprachoroidal hemorrhage requiring urgent drainage. Furthermore, the only difference in technique between needling performed in the clinic vs. the OR was releasing posterior adhesions of the bleb in the OR. Our study suggests that this extra step did not yield any improvement in success. Releasing posterior adhesions has a higher risk of injuring posterior blood vessels that have a high tendency to bleed and may increase the risk of fibrosis, so we would recommend not pursuing posterior adhesions based off the results of our study.

Secondary analysis revealed no difference of needling outcomes in patients with prior trabeculectomy, ExPress shunt, and XEN gel stent. However, baseline characteristics were different. Baseline IOP was similar between the procedure groups, but patients with XEN gel stent were on significantly fewer medications prior to bleb needling (1.1 ± 1.3, *P* = 0.03). The average time from the surgery to bleb needling was also significantly shorter with XEN (1.7 ± 1.7 months, *P* < 0.001). This may be explained by the fact that XEN gel stent is a newer procedure and reflects the authors’ beliefs that needling after XEN may be more effective when performed early in the postoperative period before adding back multiple medications. This also explains the higher than average bleb needling rate for XEN gel stent in this study (53.1% in this study vs. up to 44.1% in the existing literature [4, 10–12]). This is in contrast to patients with failed trabeculectomy or ExPress shunt who are more likely be treated with maximum tolerable medications, often by outside providers, before proceeding with bleb needling given concerns for higher risk of hypotony and vision-threatening complications [13]. Furthermore, bleb needling of XEN gel stent on average had led to equivalent final IOP and number of medications compared to bleb needling of trabeculectomy and ExPress shunt (Supplemental Table 2). The results of the multinomial logistic regression show XEN gel stent has a high odds (6.09) of success when baseline IOP, baseline medications, and needling location are controlled for compared to trabeculectomy. Though this finding was not statistically significant, a difference may be appreciated with a larger sample size.

There were nine patients in our study that had repeat bleb needling (5 in clinic and 4 in the OR) within 3 months. Of these patients only one who had a repeat needling of XEN gel stent in the clinic was able to maintain a successful outcome at the 6-month time point. Five of nine repeated bleb needling procedures were failures at 1 week follow up, and they received alternative interventions to lower IOP. Three patients that had initially received bleb needling in the clinic were repeated in the OR, and all of these cases failed by 1 month following the OR needling. These findings suggest that repeating bleb needling may not be efficacious and repeating failed clinic bleb needling in the OR may not improve outcomes.

The limitations of our study are the retrospective non-randomized design and small sample size that limited subgroup analysis. Furthermore, there may be sampling bias from the individual circumstances of each patient that led the physician to choose bleb needling in the clinic vs. the OR. Also, the population of this sample was 85% Caucasian, which does not reflect the typical mixture of patients seen by an average ophthalmologist. However, to the best of our knowledge, this is the first study that analyzed various outcome measures after bleb needling performed in the clinic vs. the OR.

## Conclusions

Our study shows that bleb needling with MMC can be effectively and safely performed in the clinic following failed trabeculectomy, ExPress shunt, or XEN gel stent. Future studies will involve a larger sample size and a prospective randomized study design comparing predictive factors and outcomes of needling after various bleb-forming procedures to better elucidate the best way to manage a failed bleb, an often unavoidable outcome of any bleb-forming procedure.

## Supplementary Information

**Additional file 1: Table S1.** Bleb revision success rate after > 1 needling procedures at each time point.

**Additional file 2: Table S2.** Mean IOP and number of medications.

**Additional file 3: Table S3.** Multinomial logistic regression for 6 month success.

**Additional file 4: Table S4:** Reneedling rate.

**Additional file 5.**

## Data Availability

All data generated or analyzed during this study are included in this published article [and its supplementary information files].

## Abbreviations

OR: Operating room
MMC: Mitomycin C
IOP: Intraocular pressure
VA: visual acuity
