# Supplementary material for: Comparative outcome analysis of bleb needling of fibrotic blebs in the clinic versus the operating room: a retrospective case series
Source: BMC Ophthalmol. 2021 Mar 4;21:115. doi: 10.1186/s12886-021-01870-1 (PMC7934488; doi:10.1186/s12886-021-01870-1)
Supplement: Supplementary file 1 — Additional file 1: Table S1. Bleb revision success rate after > 1 needling procedures at each time point. [file 12886_2021_1870_MOESM1_ESM.pdf]

Supplemental Table 1: Bleb revision success rate after > 1 needling procedures at each time point.

	**1w**	**1m**	**2m**	**6m**
Clinic	40% (2/5)	20% (1/5)	20% (1/5)	20% (1/5)
OR	50% (2/4)	50% (2/4)	0% (0/4)	0% (0/4)
Trabeculectomy	33% (1/3)	33% (1/3)	0% (0/3)	0% (0/3)
ExPress	50% (1/2)	50% (1/2)	0% (0/2)	0% (0/2)
XEN	50% (2/4)	25% (1/4)	25% (1/4)	25% (1/4)

Abbreviations: w = week, m = month(s)
