# Supplementary material for: Comparative outcome analysis of bleb needling of fibrotic blebs in the clinic versus the operating room: a retrospective case series
Source: BMC Ophthalmol. 2021 Mar 4;21:115. doi: 10.1186/s12886-021-01870-1 (PMC7934488; doi:10.1186/s12886-021-01870-1)
Supplement: Supplementary file 2 — Additional file 2: Table S2. Mean IOP and number of medications. [file 12886_2021_1870_MOESM2_ESM.pdf]

Supplemental Table 2: Mean IOP and number of medications

**Mean IOP (SE)**	**1w**	**1m**	**2m**	**6m**
Clinic	15.4 (1.6)	16.3 (1.2)	14.7 (1.2)	14.2 (0.8)
OR	15.4 (2.1)	17.5 (1.9)	17.5 (1.8)	14.9 (1.8)
P-value	0.990	0.595	0.212	0.731
Trabeculectomy	14.7 (2.2)	17.8 (2.5)	15.1 (1.8)	13.4 (0.9)
ExPress	15 (1.8)	15.4 (2.1)	16.2 (2.3)	14.5 (1.4)
XEN	16.2 (2.5)	16.9 (1.4)	16.3 (1.5)	15.1 (1.2)
P-value	0.871	0.711	0.883	0.688
**Mean Medications (SE)**	**1w**	**1m**	**2m**	**6m**
Clinic	1 (0.3)	1.3 (0.3)	1.4 (0.3)	1.4 (0.3)
OR	0.8 (0.4)	1 (0.4)	1.5 (0.4)	1.7 (0.5)
P-value	0.674	0.628	0.829	0.586
Trabeculectomy	0.3 (0.3)	0.3 (0.3)	1 (0.5)	1.1 (0.5)
ExPress	1.1 (0.5)	1 (0.5)	1.6 (0.4)	1.4 (0.4)
XEN	1.2 (0.3)	1.7 (0.3)	1.6 (0.4)	1.9 (0.5)
P-value	0.244	0.060	0.549	0.475

Abbreviations: SE = standard error, w = week, m = month(s).
