# Supplementary material for: Comparative outcome analysis of bleb needling of fibrotic blebs in the clinic versus the operating room: a retrospective case series
Source: BMC Ophthalmol. 2021 Mar 4;21:115. doi: 10.1186/s12886-021-01870-1 (PMC7934488; doi:10.1186/s12886-021-01870-1)
Supplement: Supplementary file 3 — Additional file 3: Table S3. Multinomial logistic regression for 6 month success. [file 12886_2021_1870_MOESM3_ESM.pdf]

Supplemental Table 3: Multinomial logistic regression for 6 month success

	B	Odds Ratio	SE	P-value	Lower Bound	Upper Bound
Intercept	-0.314		1.555	0.840		
Baseline IOP	0.039	1.040	0.053	0.461	0.937	1.155
Baseline Medications	0.246	1.279	0.251	0.328	0.782	2.092
Clinic (compared to OR)	-0.771	0.462	0.913	0.398	0.077	2.769
ExPress (compared to Trabeculectomy)	0.056	1.058	0.894	0.736	0.184	2.092
XEN (compared to Trabeculectomy)	1.806	6.087	1.159	0.119	0.628	58.968

Abbreviations: IOP = intraocular pressure, SE = Standard error
