# Supplementary material for: Comparative outcome analysis of bleb needling of fibrotic blebs in the clinic versus the operating room: a retrospective case series
Source: BMC Ophthalmol. 2021 Mar 4;21:115. doi: 10.1186/s12886-021-01870-1 (PMC7934488; doi:10.1186/s12886-021-01870-1)
Supplement: Supplementary file 4 — Additional file 4: Table S4: Reneedling rate. [file 12886_2021_1870_MOESM4_ESM.pdf]

**Supplemental Table 4:** Reneedling rate[a]	
Clinic	25% (8/32)
OR	7% (1/15)
	P = 0.136
Trabeculectomy	22% (2/14)
ExPress	13% (2/16)
XEN	18% (3/17)
	P = 0.915
XEN Needling Rate	53.1% (17/32[b])

[a]Rate of repeat bleb needling events within 3 months of most recent needling.
[b]Total of all XEN gel stents performed within study dates that required needling.
